# *Pasteurella multocida* Bacteremia and Peritonitis in a Patient With Cirrhosis: A Life-Threatening Case From a Prick of a Cactus

**DOI:** 10.1177/2324709617726103

**Published:** 2017-08-30

**Authors:** Jodi-Anne Wallace, Jonathan Hussain, Alberto Unzueta, Giuseppe Morelli

**Affiliations:** 1 University of Florida, Gainesville, FL, USA

**Keywords:** cactus, cirrhosis, multocida, *Pasteurella*, peritonitis

## Abstract

A 58-year-old male with nonalcoholic steatohepatitis cirrhosis presents with right lower extremity cellulitis, abdominal tenderness, and severe sepsis after sustaining puncture injury from a cactus on a property with feral cats. Blood cultures and diagnostic paracentesis were consistent with spontaneous bacterial peritonitis due to *Pasteurella multocida*, a gram-negative coccobacillus found in the respiratory tract of domestic animals. The patient received timely antibiotic coverage with resolution of spontaneous bacterial peritonitis and sepsis after 14-day treatment. This case emphasizes the life-threatening nature of systemic *Pasteurella multocida* infection as well as an indirect way of acquiring a zoonotic infection in a patient with end-stage liver disease.

## Background

We report this case to describe an uncommon presentation of *Pasteurella multocida* peritonitis due to indirect exposure in a patient with end-stage liver disease. This presentation emphasizes the impact of systemic infection in this immunocompromised population and stresses the importance of timely initiation of broad-spectrum antibiotics in severe sepsis.

## Case Presentation

A 58-year-old male with a past medical history significant for nonalcoholic steatohepatitis cirrhosis (Child-Pugh Class C; Model for End-Stage Liver Disease score 20) that was on liver transplant waiting list presented with right lower extremity cellulitis, abdominal pain with distention, and severe sepsis 3 days after sustaining a puncture wound from a cactus. He reported general malaise, subjective fevers, abdominal pain with distention, new bilateral lower extremity edema, and new right lower extremity erythema near the puncture within 48 hours of his initial injury. He first visited his primary care physician who directed him to the emergency department for hypotension of 99/50 mm Hg. He was subsequently found to be tachycardic (101 beats per minute), tachypneic (26 breaths per minute), and normotensive (118/64 mm Hg) after receiving 2 liters of normal saline. On examination, he had a warm, petechial rash extending along the medial aspect of the right lower extremity, abdominal distention, and 3+ pitting edema to the thigh bilaterally.

## Investigations

Initial laboratory evaluation was remarkable for leukocytosis with white blood cell count of 10 000/µL with 22% bands. Basic metabolic panel revealed sodium of 130 mEq/L, bicarbonate of 13 mEq/L, urea nitrogen of 22 mg/dL, creatinine of 1.41 mg/dL (baseline of 0.8 mg/dL), and glucose of 63 mg/dL. Tests for liver function showed elevation of total bilirubin at 6.2 mg/dL and international normalized ratio at 3.2. Blood cultures were obtained on admission prior to antibiotic administration. Diagnostic paracentesis was performed revealing a cell count of the ascites fluid significant for 8900/µL white blood cells with 86% neutrophils. A computed tomography scan of the right lower extremity revealed diffuse fluid and tissue swelling throughout the superficial soft tissues that did not cross fascial planes consistent with right lower extremity cellulitis. The patient was also noted on echocardiogram to have a decrease in left ventricular ejection fraction from 60% to 40%.

## Differential Diagnosis

Given the diagnostic paracentesis findings, it was clear the patient had spontaneous bacterial peritonitis (SBP). However, the underlying causative pathogen remained unclear. As a result, the differential diagnosis included SBP from common anaerobic bacteria, like *Escherichia coli*, versus SBP from gram-positive organisms that often cause cellulitis, like streptococcus. Blood cultures ultimately resulted with *Pasteurella multocida*, which in combination with peritoneal fluid analysis confirmed the diagnosis of SBP due to *Pasteurella multocida*.

## Treatment

The patient was admitted to the intensive care unit for severe hypoglycemia and sepsis. He received 6 liters of normal saline resuscitation and was started on a dextrose 10% infusion until blood glucose normalized. He was placed on broad-spectrum antibiotics including vancomycin, ceftazidime, and metronidazole. A diagnostic paracentesis was performed, and he was subsequently treated with albumin as per SBP guidelines protocol (1.5 mg/kg day on day 1 and 1 mg/kg on day 3).^1^ The antibiotics were narrowed to ceftriaxone and later amoxicillin for a 14-day total of antibiotics based on culture sensitivities. His cellulitis was complicated by underlying lower extremity edema due to chronic diastolic heart failure and cirrhosis. He required intravenous diuretics, with improvement of the cellulitis as his edema improved.

## Outcome and Follow-up

The patient is currently listed for liver transplant, he has not had further infectious disease complications, and his Model for End-Stage Liver Disease score is 19. He still has lower extremity edema and is on diuretics, and his decreased left ventricular ejection fraction has returned to normal.

## Discussion

*Pasteurella multocida* is a zoonotic anaerobic gram-negative coccobacillus, often found in the respiratory tracts of domestic animals, like dogs and cats. It is the most infectious species of the *Pasteurella* genus and has been isolated in 25% of dog bites and 60% to 80% of cat bites.^2,3^ The documented *P multocida* infections are often a result of direct bites or scratches from domestic pets that present as cellulitis or septic arthritis. Disseminated *P multocida* infection has been noted to carry a 20% to 30% mortality rate, and can cause severe infection in healthy individuals.^4,5^ Life-threatening infections have been reported in immunocompromised patients including infective endocarditis and sepsis.^6,7^ There are few published cases of SBP due to *P multocida* infection. SBP is typically caused by translocation of gram-negative anaerobic bowel bacteria, like *E coli*, into the peritoneal fluid. Severe intraabdominal infection by *P multocida* often occurs in patients with cirrhosis or who are undergoing peritoneal dialysis. Like the general population, these cases are often the result of direct insult from pets. Of the cirrhotic cases, the outcomes have ranged from complete resolution to death.^8,9^

Quite rare there are a few documented accounts of indirect exposures to animals that resulted in severe *P multocida* infection in patients with cirrhosis. Of those, patients presented with bacteremia, arthritis, pneumonia, or peritonitis.^10-14^ The general theory behind many these cases is nasopharyngeal colonization by frequent exposure to domestic animals that lead to systemic infection when trauma occurs to the nasopharynx or oropharynx. The nature of the trauma can vary from head and neck radiation to upper gastrointestinal endoscopy.^11,13^ Another case suggests an alternative theory of human gastrointestinal colonization of *P multocida* that translocated to the peritoneal fluid to cause peritonitis.^8^ However, direct testing for colonization of that patient was not performed. In addition, there are no published reports of *P multocida* colonization of the skin. Rather, normal skin flora often includes gram-positive organisms like *Streptococcus* and *Staphylococcus* species. Likewise, the presence of *Pasteurella* species on cactus thorns has not been documented.

There are no specific guidelines for the treatment of *P multocida* peritonitis, and empiric coverage for *P multocida* should include amoxicillin-clavulanate for patients that can tolerate an oral regimen and have mild infection.^15^ If unable to take oral medications, ampicillin-sulbactam should be used in mild infection. Broader spectrum like piperacillin-taxobactam, a third-generation cephalosporin with metronidazole or a carbapenem, should be used in severe infections that include bacteremia, deep tissue infection, or respiratory infection. Bacteremia is generally treated for 14 days after first negative culture.

After further history, it was revealed that our patient has multiple feral cats that roam his property. He denied ever having direct contact with these cats, but it was concluded that these cats likely had passed near the cacti prior to his injury. It is uncertain the timeline or the nature of the feline exposure, but within 24 hours of the injury he became symptomatic. It is uncertain if the patient has *P multocida* colonization, but he has no known insult to his nasopharynx or oropharynx and had not undergone upper gastrointestinal endoscopy recently. Due to his septic presentation, he was started on broad-spectrum antibiotics for gram-positive and gram-negative coverage until blood cultures revealed *P multocida* sensitive to ceftriaxone and amoxicillin. This unique case reports a new indirect exposure to *P multocida* that resulted in severe peritoneal infection. It reminds of how quickly and susceptible patients with cirrhosis can contract life-threatening infection and the importance of educating this population on exposure risks. It also shows the importance of obtaining cultures and the timely initiation of broad-spectrum antibiotics in patients with sepsis.
